# What are the beliefs of pediatricians and dietitians regarding complementary food introduction to prevent allergy?

**DOI:** 10.1186/1710-1492-7-S2-A3

**Published:** 2011-11-14

**Authors:** Sara H Leo, John M Dean, Edmond S Chan

**Affiliations:** 1 Department of Pediatrics, Division of Allergy, University of British Columbia, Vancouver, British Columbia, Canada, V6H 3V4; 2 BC Children’s Hospital, Vancouver, British Columbia, Canada, V6H 3V4

## Background

Food allergy often manifests on first known oral exposure. Hence, the timing of complementary food introduction is of interest. The American Academy of Pediatrics offers specific dietary guidelines that were updated in 2008.

Objective: We wanted to identify the recommendations that general pediatricians and registered dietitians provide to parents and delineate any differences in counselling.

## Methods

A 9-item survey was distributed to pediatricians and dietitians online and by mail. Information on practitioner type, gender, length of practice and specific recommendations made regarding complementary food introduction and exposure was collected.

## Results

181 surveys were returned with a 54% response rate from pediatricians. 52.5% of all respondents were pediatricians and 45.9% were dietitians. The majority of pediatricians and dietitians advise mothers that peanut abstinence during pregnancy and lactation is unnecessary. Dietitians were more likely to counsel mothers to breastfeed their infants to prevent development of atopic dermatitis than pediatricians. Hydrolyzed formulas for infants at risk of developing allergy were the top choice of formula amongst both practitioners. Pediatricians were more likely to recommend delayed introduction of peanut and egg, while most dietitians recommended no delay in allergenic food introduction to prevent development of food allergy.

## Conclusions

With the exception of whether to recommend breastfeeding to prevent development of atopic dermatitis and whether to delay allergenic food introduction, pediatricians and dietitians agreed closely in their advice and adhered to the 2008 American Academy of Pediatric guidelines. Further education using the latest recommendations should be considered.

